# Presenilin1 inhibits glioblastoma cell invasiveness via promoting Sortilin cleavage

**DOI:** 10.1186/s12964-021-00780-5

**Published:** 2021-11-15

**Authors:** Wei Yang, Yan Xiang, Mao-Jun Liao, Peng-Fei Wu, Lin Yang, Guo-Hao Huang, Bao-Zhong Shi, Liang Yi, Sheng-Qing Lv

**Affiliations:** 1 Department of Neurosurgery, Xinqiao Hospital, Army Medical University, 183# Xinqiao street, Shapingba District, Chongqing, 400037 China; 2 Department of Neurosurgery, Daping Hospital, Army Medical University, 10# Changjiangzhi Road, Daping, Yuzhong District, Chongqing, 400042 China; 3 Department of Critical Care Medicine & Department of Neurosurgery, The First Affiliated Hospital & College of Clinical Medical, Henan University of Science and Technology, Luoyang, 471003 Henan China

**Keywords:** Presenilin1, Cleavage, Sortilin, Glioblastoma, Invasion

## Abstract

**Background:**

Alzheimer’s disease (AD) and glioblastoma are the most common and devastating diseases in the neurology and neurosurgery departments, respectively. Our previous research reports that the AD-related protein Presenilin1 represses cell proliferation by inhibiting the Wnt/β-catenin pathway in glioblastoma. However, the function of Presenilin1 and the underlying mechanism need to be further investigated.

**Methods:**

The correlations of two genes were conducted on the R2 microarray platform and CGGA. Wound healing, Transwell assays and glioblastoma transplantation were performed to detect invasion ability. Phalloidin staining was employed to show cell morphology. Proximity ligation assays and protein docking assays were employed to detect two protein locations. We also employed western blotting to detect protein expression.

**Results:**

We found that Presenilin1 clearly repressed the migration, invasion and mesenchymal transition of glioblastoma cells. Intriguingly, we observed that the expression of Presenilin1 was positively correlated with Sortilin, which is identified as a pro-invasion molecule in glioma. Furthermore, Presenilin1 interacted with Sortilin at the transmembrane domain and repressed Sortilin expression by cleaving it in glioblastoma cells. First, we found that Sortilin introduced the function of Presenilin1 in phosphorylating β-catenin and repressing invasion in glioblastoma cells. Last, Presenilin1 stimulation sharply suppressed the invasion and mesenchymal transition of glioblastoma in mouse subcutaneous and intracranial transplantation models.

**Conclusions:**

Our study reveals that Sortilin mediates the regulation of β-catenin by Presenilin1 and transduces the anti-invasive function of Presenilin1, which may provide novel therapeutic targets for glioblastoma treatment.

**Video Abstract**

**Supplementary Information:**

The online version contains supplementary material available at 10.1186/s12964-021-00780-5.

## Background

Glioma is the most common and lethal primary brain tumor in the central nervous system. The poor prognosis of glioma patients is mainly due to the highly invasive character of these tumor cells [1]. Tumor cells invading the parenchyma lead to incomplete surgical resection and are extremely resistant to chemoradiotherapy; however, there are currently no anti-invasive therapies available in the clinic [2]. Therefore, it is imperative to understand the detailed molecular pathogenesis of invasion and develop novel therapeutic strategies.

The Presenilin1 (PS1) protein is encoded by the PSEN1 gene, which is located on chromosome 14 in humans, and has been well investigated in Alzheimer’s disease (AD) [3]. This protein is a key catalytic component of the γ-secretase complex, which contains nicastrin (NCT), anterior pharynx-defective 1 (Aph-1), and presenilin enhancer 2 (Pen-2), responsible for the intramembranous cleavage of amyloid precursor protein (APP). The dysfunction of the Presenilin1 gene leading to aberrant APP cleavage is a central process in AD pathogenesis [4]. In 2002, Kang DE et al. reported that Presenilin1 contributes to β-catenin degradation by facilitating the stepwise phosphorylation of β-catenin independent of the Axin complex in embryogenesis and tumorigenesis [5]. Sequentially, Presenilin1 was reported to be involved in several kinds of cancer except glioma [6]. Our research demonstrated that Presenilin1 exerts antiproliferative effects on glioblastoma cells by repressing β-catenin [7]. However, the regulatory mechanism of Presenilin1 on β-catenin is not well illuminated, and the function of Presenilin1 in glioma also needs to be further investigated.

Coincidentally, Sortilin belongs to the new type 1 single transmembrane receptor family, which also includes sorting protein-related receptor with A-type repeats (SorLA) and Sortilin-related receptor central nervous system expressed 1 (SorCS 1), SorCS2 and SorCS3 [8]. Recently, Sortilin was identified as an important molecule that participates in the development of both amyloid and tau pathologies. In particular, Sortilin degrades proteins and generates insoluble peptidic fragments that deposit on neuritic plaques [9]. On the other hand, Sortilin also plays important biological roles in carcinomas and therefore the prognosis of cancer patients, including glioma [10]. We found that Sortilin is highly expressed and involved in the stable expression and nuclear accumulation of β-catenin, but Presenilin1 represses the stability and enhances the degradation of β-catenin, regulating glioblastoma aggressiveness [7, 11]. Previous reports suggest that Presenilin1 participates in the cleavage of type 1 single transmembrane proteins, such as CD44, Notch, Delta, Jagged, E- and N-cadherin [12–16]. Recently, Sortilin has been shown to be a substrate for Presenilin1-dependent γ-secretase [17, 18]. It is interesting to investigate whether Presenilin1 could interact with Sortilin and determine its relative biofunction in glioblastoma.

In this study, we report that Presenilin1 represses the migration, invasion and mesenchymal transition ability of glioblastoma cells. Furthermore, the expression level of preseinlin1 maintains a positive correlation with that of Sortilin in glioma patients, and Presenilin1 could interact and cleave Sortilin. The cleavage of Sortilin leads to the suppression of β-catenin, which hinders mesenchymal transition and invasion of glioblastoma cells. These results suggest that Presenilin1 has significant roles in the anti-invasive behavior of glioblastoma through the cleavage of Sortilin, and stimulation of Presenilin1 may be a new strategy for glioblastoma treatment.

## Materials and methods

### Cell culture

Human glioblastoma cell lines (U87 and U251) were purchased from the American Type Culture Collection (ATCC;USA). The STR identification of cell lines was conducted with Applied Biosystems. All cells were cultured with DMEM/F12(Hyclone, USA) supplemented with 10% fetal bovine serum (FBS; Gibco), 1% penicillin and streptomycin (Beyotime)in a humidified incubator at 37 °C with 5% CO_2_.

### Reagents and antibodies

AF38469 and RO4909497 was purchased from Med Chem Express. Lipofectamine 2000 was obtained from Invitrogen. FITC-phalloidin was purchased from Life Technological. DMSO and DAPI were obtained from Sigma Aldrich. Mouse anti-Presenilin1 monoclonal antibody was purchased from CST. Rabbit anti-Sortilin monoclonal antibody was purchased from GeneTex. β-Catenin Antibody Sampler Kit, Anti-N-cadherin, Vimentin, MMP-2 and Actin antibodies were purchased from CST.

### Analysis of patient data

Kaplan–Meier analysis was conducted with the mRNA_array_301 dataset from CGGA (Chinese Glioma Genomics Atlas, http://www.cgga.org.cn), which includes 262 primary glioma cases and 22 recurrent glioma cases, respectively. We analyzed the relations of Presenilin1 and EMT-markers (Twsit1, Twsit2, Zeb1, Zeb2, Slug1 and Slug2) at R2 Genomics Analysis and Visualization Platform (RGAVP, https://hgserver1.amc.nl/cgi-bin/r2/main), and “Tumor Glioma-French-284-MAS5.0-u133p2” was selected for analyzed. “Tumor Glioma-French-284-MAS5.0-u133p2”, “Tumor Glioma-Freiji-85-MAS5.0-u133a” and “Tumor Glioblastoma-TCGA-540- MAS5.0-u133a” were utilized to analysis the correlation of Presenilin1 and Sortilin. On the other hand, the correlation of Presenilin1 with Sortilin was also analyzed in mRNAseq_325 dataset, mRNAseq_693 dataset and mRNA_array_301 dataset on CGGA. The Rand *p* values of linear regression were downloaded from the Internet.

### Protein interaction analysis

The interaction of Sortilin and related protein was conducted on pathway commons platform (http://www.pathwaycommons.org). We selected the other 25 proteins which divided into binding and modification groups.

### Lentivirus transfection

We obtained the PSEN1 stable silence and over-expression cell lines as previous described [7]. Particularly, lentiviral containing with SORT1 sequence (Lv-SORT1) was constructed by Genechem (Shanghai, China).

### Cell migration and invasion assays

Stable silence or over-expression of PSEN1 Cells and control cells were seeded in 6-well plates, after reached at 90% confluence and starved for 8 h. A wounding line was scratched with 10 μl pipet tip, and the serum-free medium was added to the plates. Cells were incubated for 24 h. The migrated cells were monitored with Olympus inverted microscope and counted in 5 randomly selected fields to quantify the cell migration area by Image J.

For detecting invasion ability, Transwell filters (8.0 μM pore size, Corning) were pre-coated with Matrigel (Corning). The transfected cells were starved and 5 × 10^4^ cells in 100 μl of serum-free medium were seeded into the upper chamber. The lower chamber was filled with 600 μL of medium containing 5% fetal bovine serum. After 24 h of incubation, cells were removed from the upper surface. Invaded cells on the lower surface were fixed, permeated, and stained with 5% crystal violet (Beyotime). Invaded cells were counted in 5 randomly selected fields to quantify the invasive rate.

### HE staining and phalloidin staining

Mouse transplanting GBM tissues were fixed and cut into 5 μm sections, and preformed HE staining by all-in-one machine. Cells were planted in confocal dish and cultured for 24 h, then fixed and permeated, stained with FITC-phalloidin (1:100) overnight. Washed with PBS for three times and imaged on co-focal laser scanning microscope (Leica TCS SP8, Germany).

### Immunohistochemistry (IHC) and immunofluorescence (IF)

Human glioblastoma tissues were fixed and cut into 5 μm sections. The slides were treated for antigen retrieval, incubated with primary (Presenilin1 1:100) and secondary antibodies, and enzyme conjugate horseradish peroxidase. For IF, the slides were fixed and blocked, then permeated and incubated with primary antibodies (Sortilin 1:100; Nestin 1:200) at 4 °C overnight and incubated with Fluorescence labeled second antibodies (1:200). The nucleus was stained with DAPI and examined with co-focal laser scanning microscope (Leica TCS SP8, Germany). The images were processed by LAS AF Lite software (Leica, Germany).

### Western blotting assay

The total protein was extracted using RIPA lysis buffer (Beyotime, Shanghai, China) with 1% PMSF (Beyotime) and 1 × Phosphorylase inhibitors (Solabio, Beijing, China), and BCA kit (Beyotime) was used to measure the concentration according to the manufacturer’s instructions. The proteins were separated by SDS-PAGE gel electrophoresis and transferred to PVDF membrane (Millipore). The membrane was blocked and incubated with primary antibodies (Presenilin1,1:2000; Sortilin,1:2000; β-Catenin,1:2000; p45-β-Catenin,1:2000; p33/37/41-β-Catenin,1:2000; N-cadherin, 1:1000; MMP2,1:2000; Vimentin, 1:2000; Actin,1:5000) and the IgG-hydrogen peroxide (HRP) secondary antibody in sequence. The protein blots were exposed to Clarity™ Western ECL substrate (BIO-RAD) using the BIO-RAD ChemiDoc™ Touch Imaging System. The gray value was detected by Image J.

### Proximal ligation assay (PLA)

4 × 10^4^ glioma cells/dish were plated on confocal culture dish and incubated overnight, cells were fixed with 4% paraformaldehyde for half hour and permeabilized with 0.03% TritonX-100 for 10 min at room temperature. Washed dishes with 1X PBS three times, then blocked for 30 min at 37 °C with 5%BSA. Added enough of both primary antibodies (rabbit anti-Sortilin monoclonal antibody 1:100, mouse anti-Presenilin1 monoclonal antibody 1:100) and incubated overnight at 4 °C. Washed dishes with Buffer A (Sigma-Aldrich) two times for five minutes each on shaker at room temperature. Added oligonucleotide-linked secondary antibody solution (Duolink® in situ PLA® probe anti-mouse MINUS 1:10; Duolink® in situ PLA® probe anti-rabbit PLUS 1:10; Sigma-Aldrich) and Incubated in a humidity chamber for 1 h at 37 °C. After washed two times with Buffer A, added ligase solution to dishes (Duolink® in situ detection reagents red, Sigma-Aldrich) and incubated for 30 min in a humidity chamber at 37 °C. Washed two times. Added premixed polymerase and nucleotide solution (Duolink® in situ detection reagents red, Sigma-Aldrich) and incubated for 100 min at 37 °C. Washed with wash Buffer B (Sigma-Aldrich) for two times. Dishes were counterstained with DAPI to visualize cell nuclei. Imaged the fluorescence on co-focal laser scanning microscope (Leica TCS SP8, Germany) and quantified the PLA reaction with Image J software.

### Protein docking assays

The crystal structures of Presenilin1and Sortilin were retrieved from the MICOS Data Bank, the blind binding modes of preseinlin1 with Sortilin was predicted by Rosetta docking software and got 100 docking phases of every system. Then we chose the lowest docking energy phase for next assays. The molecular dynamics analyses were conducted by Amber software, time was last 100 ns and the RMSD value was stabilized at 60 ns, and we chose the average structure phase during 60 ns to 100 ns for detail docking model analyses. And we obtained the docking amino acids and detail feature.

### Tumor xenografts

All experimental procedures were approved by the Institutional Animal Care and Use Committee of Third Military Medical University. Four-week-old mice were randomly divided into two groups (n = 4 per group), cells previously transfected with Presenilin1 overexpressing lentivirus or negative control lentivirus, and accounted 1 × 10^6^ cells per position. Cells were mixed with Matrigel (50% volume) and implanted subcutaneously into the right and left inguinal folds of the nude mice. Tumor volume was determined using an external caliper and calculated by using the Formula = (Length × Width^2^)/2. Mice were sacrificed 21 days after implantation, and tumors were excised and subjected to next Western-Blot analyses. For the orthotropic model, 1 × 10^5^ U87-Lv-Ctr and U87-Lv-PS1 cells were injected into the right corpus striatum of the brains of 6 weeks-old nude mice using a stereotactic frame. Recorded the survival time of each mouse and used GraphPad Prism 6.0 software to calculate Kaplan-Maier survival curves. Mice were monitored and sacrificed when neurological signs appeared or after 40 days. And mouse brains were collected for imaged and following HE staining.

### Statistical analysis

All experiments were performed in triplicate. Statistical analyses were carried out by using SPSS 13.0 statistical software. All data were exhibited as means ± SD and showed as histogram created by GraphPad Prism 6.0. Statistical significance was calculated by Student’s t-test or one-way ANOVA. Survival was analyzed by Kaplan–Meier method and compared by log-rank test by GraphPad Prism 6.0. (*) *p* < 0.05 was considered statistically significant.

## Results

### Presenilin1 inhibits the migration and invasion of glioblastoma cells in vitro

**Fig. 1:**
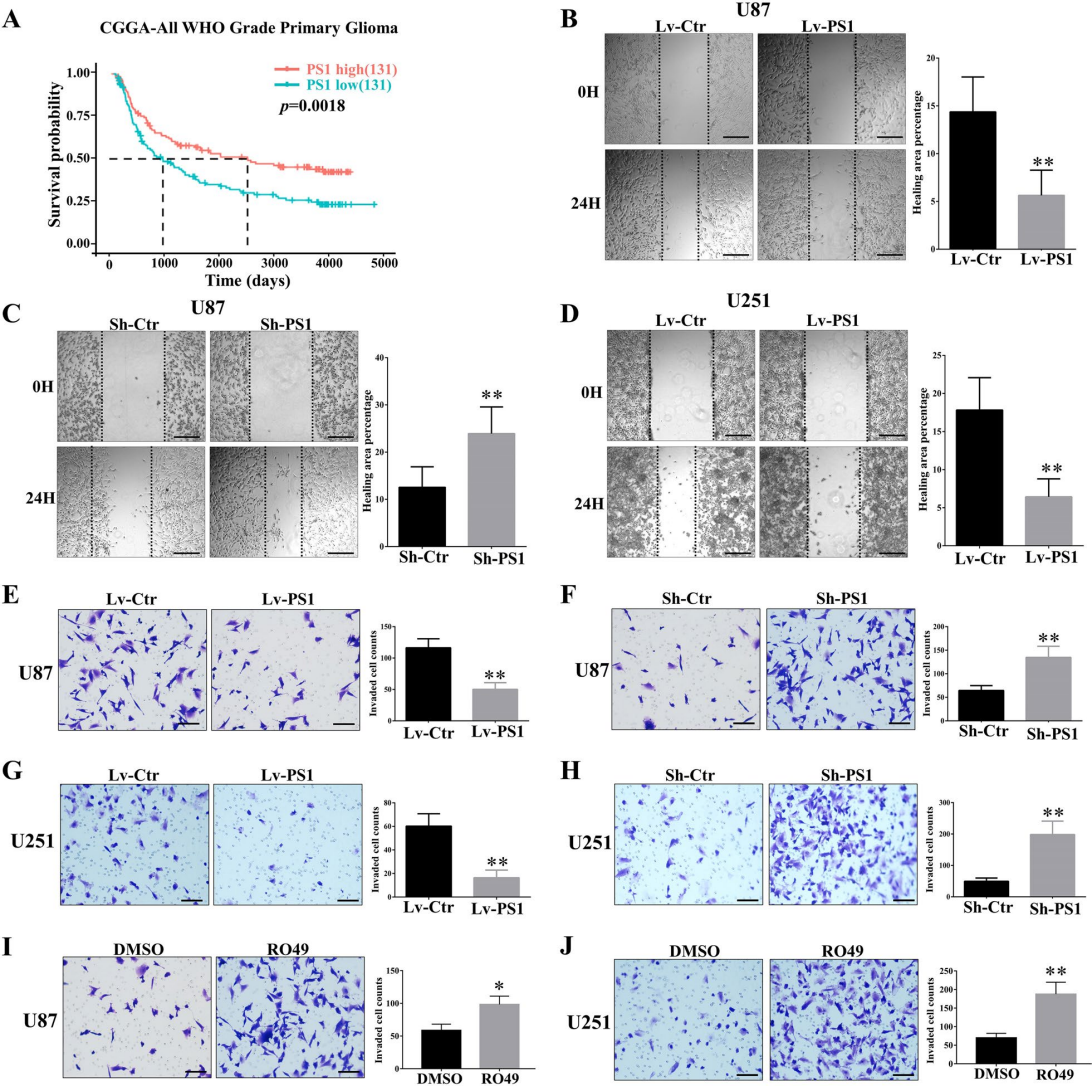
Presenilin1 inhibits migration and invasion of glioblastoma cell in vitro. **A** Kaplan–Meier analysis for all grade glioma patients. patients in the high Presenilin1 group (n = 131) and in the low Presenilin1 group (n = 131) (*p* = 0.0018, log-rank test). **B–D** Representative images of wound healing assays using U87 and U251 cells after over- or down-expression of Presenilin1. **E–H **Representative images of Transwell invasion assay using U87 and U251 cells transfected with Lv-PS1 and Sh-PS1. **I**, **J** Representative images of Transwell invasion assays using U87 and U251 cells after treated with Presenilin1 inhibitor RO4909497 (RO49). Quantification of wound healing assays were displayed on the right. Scale bar = 100 μm. **p < 0.01, *p < 0.05

First, through Kaplan–Meier analysis in CGGA, we found that the expression level of Presenilin1 maintained a significantly positive relation with patient prognosis in the Chinese primary glioma dataset, which included 262 all WHO grade glioma cases. The median survival time of the Presenilin1^high^ group was twofold longer than that of the Presenilin1^low^ group. This result was consistent with our previous results from the TCGA dataset [7] (Fig. 1A). However, Presenilin1 expression was not significantly correlated with the prognosis of recurrent glioma patients (Additional file 2: Figure S1A). Preenilin1 was barely expressed in glioblastoma tissues, which is also consistent with previous results (Additional file 2: Figure S1B**)**. To further investigate the role of presenilin in glioma, we used lentivirus to downregulate or upregulate Presenilin1 (Additional file 2: Figure S1C), then performed wound-healing assays to detect migration ability changes after loss- or gain-of-function Presenilin1, and found that upregulation of Presenilin1 significantly impaired the migratory capability (Fig. 1B). In contrast, downregulation of Presenilin1 promoted the migration ability of U87 cells (Fig. 1C). Similar results were obtained from U251 cells by wound healing assays (Fig. 1D, Additional file 2: Figure S1D). Second, we examined the effect of Presenilin1 on the invasive ability of glioblastoma cells by Transwell assays. As shown in Fig. 1E, stimulation with Presenilin1 attenuated the invasion ability of U87 cells; however, inhibition of Presenilin1 significantly increased the invasive capacities of U87 cells (Fig. 1F). To provide further evidence of the effect of Presenilin1 on glioblastoma invasion, we repeated Transwell assays in U251 cells and obtained the same trend (Fig. 1G, H). Finally, we performed Transwell assays with U87 and U251 cells after RO4909497 (RO49, Presenilin1 inhibitor) treatment, and the results showed that Presenilin1 inhibition significantly reduced invaded cells compared to the control (Fig. 1I, J). These results indicate that Presenilin1 effectively hinders the migration and invasive ability of glioblastoma cells in vitro.

### Presenilin1 represses mesenchymal transition of glioblastoma cells in vitro

**Fig. 2:**
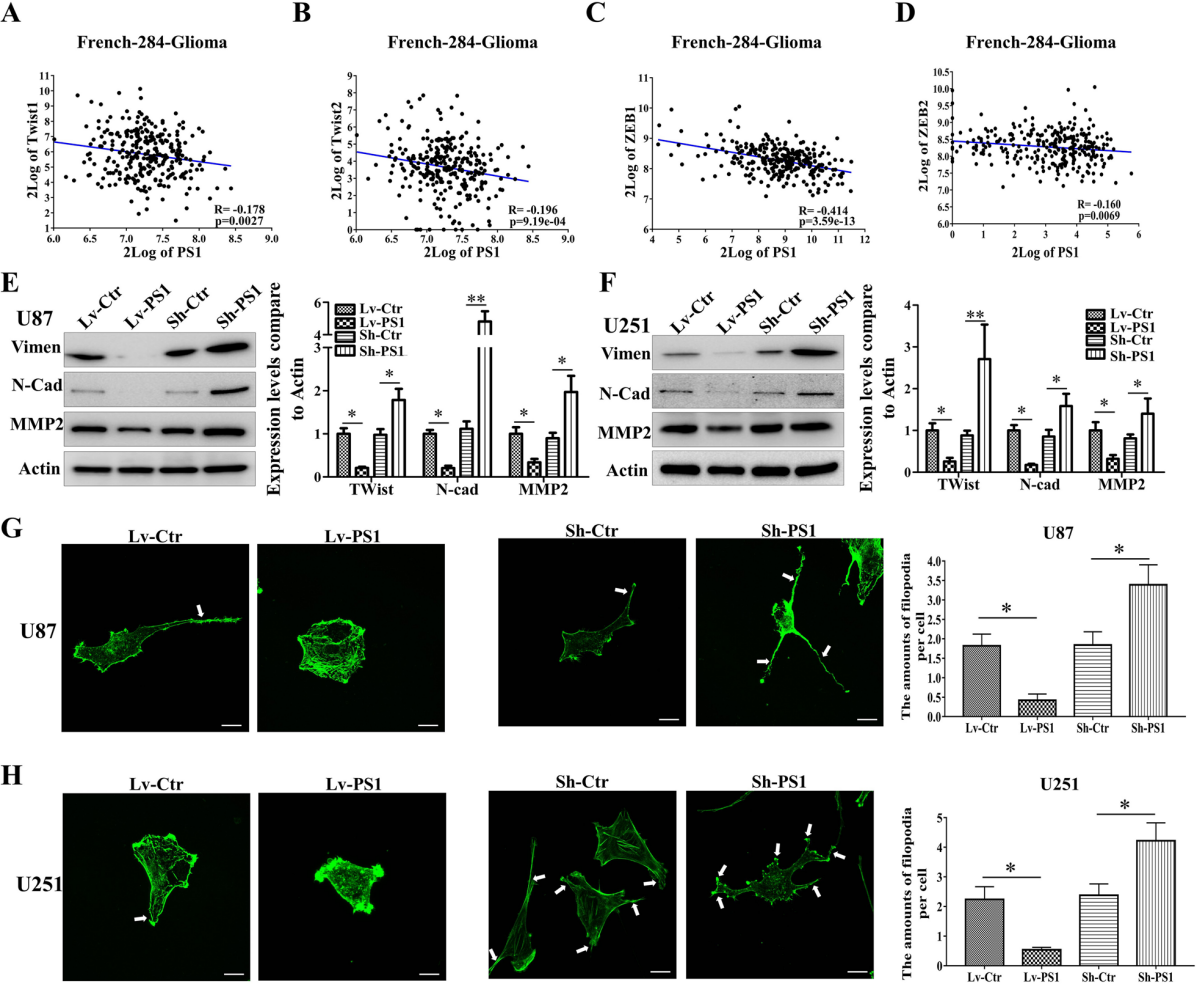
Presenilin1 represses mesenchymal transition of glioblastoma cell in vitro. **A–D** The correlations of Presenilin1 with mesenchymal transition transcription factors (Twist1, Twist2, Zeb1, Zeb2) from French-284-glioma dataset. The R and *p* values were downloaded and shown. **E**, **F** Western blot were performed to analysis the expression change of mesenchymal transition markers (Vimentin, N-cadherin and MMP2) inU87 and U251 cells when transfected with Lv-PS1, Sh-PS1 or control. **G**, **H** Representative cellular morphology change of U87 and U251 cell when transfected withLv-PS1, Sh-PS1 or control. Scale bar = 5 μm. The numbers of filopodia were calculated from five random image of each group and shown in histogram. **p < 0.01, *p < 0.05

According to previous reports, the mesenchymal transition (MT) process plays a vital function in glioblastoma cell invasion. Thus, we wondered whether Presenilin1 might inhibit invasion ability by hindering MT in glioblastoma cells. To test this hypothesis, we first analyzed the correlations between Presenilin1 expression and classical EMT-related transcription factors, such as Twist, Zeb and Slug. The results showed that the mRNA expression levels of Twist1, Twist2, Zeb1, Zeb2 and Slug2 maintained significantly negative correlations with the mRNA expression levels of Presenilin1 in gliomas of all WHO grades (Fig. 2A–D, Additional file 3: Figure S2A), but the relationships were not significant between Presenilin1 and Slug1 (Additional file 3: Figure S2B). Next, we performed western blotting assays to detect MT markers after down- or overexpression of Presenilin1 and found that the protein levels of mesenchymal markers Vimentin, N-cadherin and MMP2 were sharply decreased accompanied by Presenilin1 overexpression. In contrast, these markers were increased after downregulation of Presenilin1 in U87 cells (Fig. 2E). To further verify these results, we repeated the above assays in U251 cells and obtained a similar trend (Fig. 2F). Meanwhile, we treated U87 and U251 cells with the Presenilin-1 inhibitor RO4909497 and observed a significant increase in mesenchymal markers (Vimentin, N-cadherin, MMP-2) (Additional file 2: Figure S1E**)**. Furthermore, we utilized FITC-phalloidin staining to show the cell morphology after regulation of Presenilin1 in U87 cells. We observed that the cells became rounder and shorter from a two-polar shape after overexpression of Presenilin1. Meanwhile, a significant decrease in invasive pseudopodia was displayed after Presenilin1 overexpression, which, according to mesenchymal transition, was repressed in glioblastoma cells. Conversely, repression of Presenilin1 increased the number of dendrites and increased the length of pseudopodia, which was considered to promote MT in glioblastoma cells (Fig. 2G). Simultaneously, we confirmed the above results in U251 cells after gain- or loss-of-function of Presenilin1 (Fig. 2H). These results indicate that presenilinn1 represses the mesenchymal transition of glioblastoma cells in vitro.

### Presenilin1 interacts with Sortilin in glioblastoma cells

**Fig. 3:**
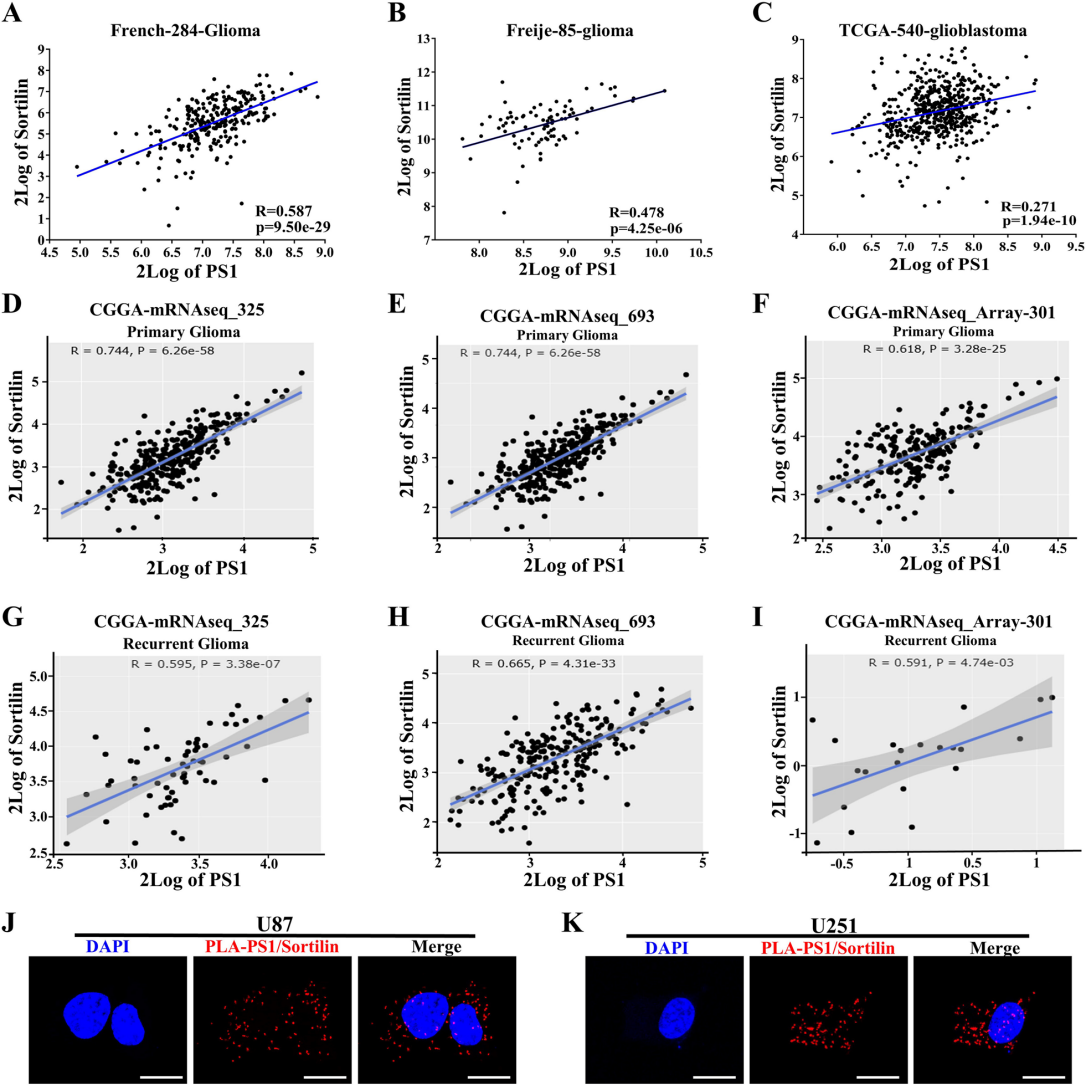
Presenilin1co-expresses and clearly co-localizes with Sortilin in glioblastoma cells. **A-C** The correlation of Presenilin1 with Sortilin from French-284-glioma dataset, Freije-85-gliomadataset and TCGA-540-glioblastoma dataset. **D–F** The relations of Presenilin1 with Sortilin frommRNAseq_325 dataset, mRNAseq_693 dataset andmRNA_array_301 dataset (contains all WHO grades primary glioma) on CGGA. **G–I** The relations of Presenilin1 with Sortilin from mRNAseq_325 dataset, mRNAseq_693 dataset andmRNA_array_301 dataset(contains all WHO grades recurrent glioma) on CGGA. **J**, **K** Proximity ligation assays (PLA) were performed on U87 and U251 cells, red spots indicate sites of PLA amplification, reflecting the Presenilin1-Sortilin co-location. Scale bar = 10 μm. The R and *p* value were downloaded and shown

Our previous research demonstrated that Sortilin promotes glioblastoma cell invasion by enhancing mesenchymal transition [11]. Because of the converse biofunction of the two proteins in glioblastoma cells, we wondered whether Presenilin1 could interact with Sortilin. First, we found that Presenilin1 could interact with Sortilin by predicting pathway commons, which implies intermodification between the two proteins (Additional file 3: Figure S2C). Next, we analyzed the expression correlation between Presenilin1 and Sortilin in glioma datasets from RGAVP, and the results showed that the mRNA level of Presenilin1 maintained a significantly positive correlation with Sortilin in glioma and GBM patients (Fig. 3A–C). Meanwhile, we observed that Presenilin1 had a positive relation with Sortilin in primary glioma patients from three datasets from CGGA (Chinese Glioma Genome Atlas) (Fig. 3D–F). Furthermore, we also found that the expression of Presenilin1 positively correlated with Sortilin expression in recurrent glioma samples (Fig. 3G–I). More detailed analysis was conducted in each WHO grade glioma, and the results illustrated that Presenilin1 expression significantly and positively correlated with Sortilin expression in every grade glioma case, whether primary or recurrent (Additional file 3: Figure S2D–F, Figure S2G–I). Next, we performed a PLA assay to detect the colocalization of Sortilin and Presenilin1 and found significant Presenilin1-Sortilin PLA signaling in U87 and U251 cells, suggesting that Presenilin1 was located in close proximity to Sortilin in GBM cells (Fig. 3J–K). These results indicate that Presenilin1 positively co-expresses and clearly colocalizes with Sortilin in glioblastoma cells.

### Presenilin1 interacts with Sortilin at the transmembrane region

**Fig. 4:**
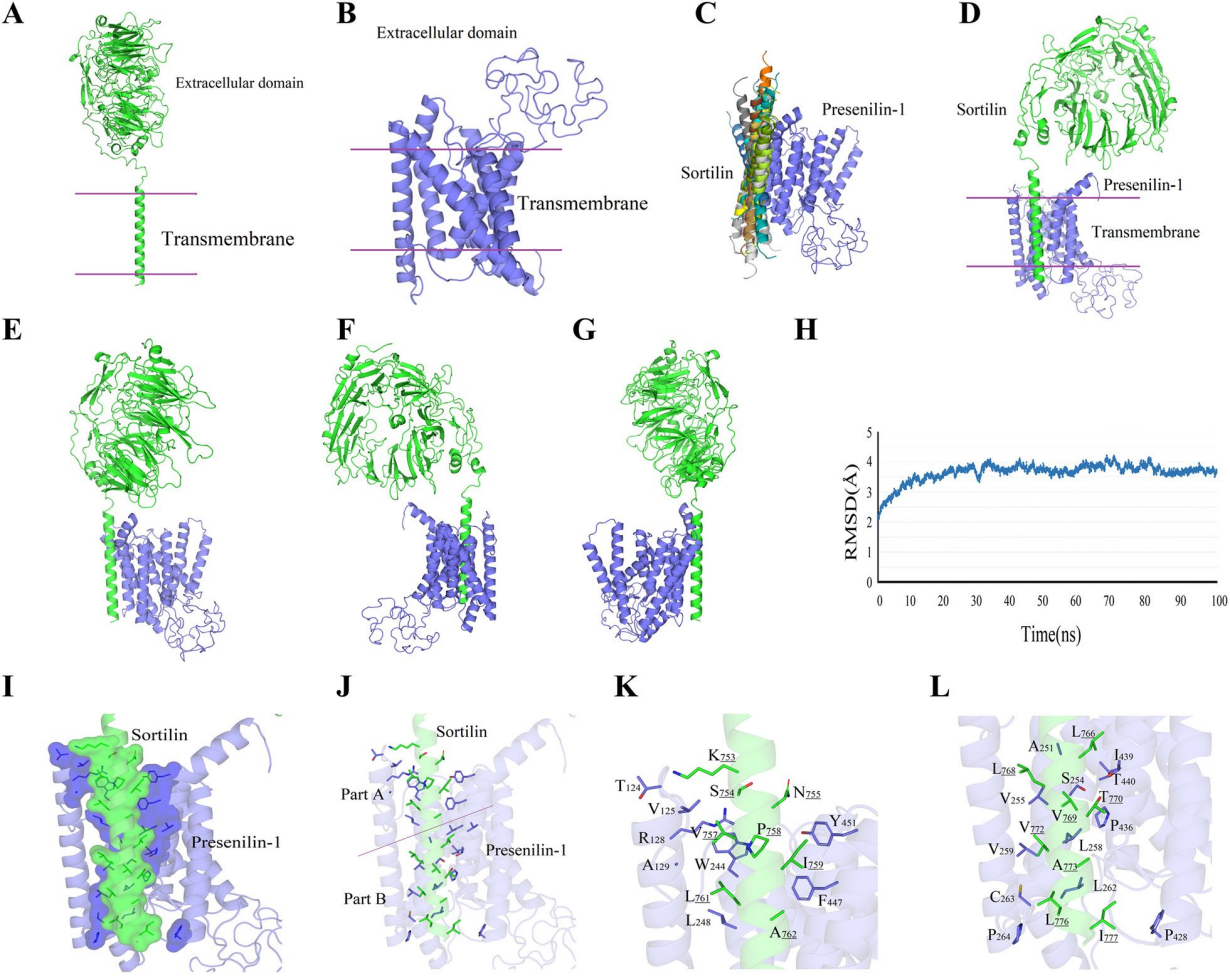
Stereo view of the docked conformation of Sortilin and Presenilin1. **A**, **B** the crystal structure of Sortilin and Presenilin1. **C** Conformational stability of Presenilin1-Sortilin docked complexes based on RMSD-score during 100 ns time period. **D–G** the different angle of the lowest docking model of Sortilin and Presenilin1. **H** The view of detail docking model for Sortilin and Presenilin1. **I** Neighbouring residues around Sortilin and Presenilin1 are represented using coloured sticks. **J**, **K** Enlarged view of the location of Part A and Part B from image J, Neighbouring residues around Sortilin and Presenilin1 are labelled

To further investigate the detailed interaction information of Presenilin1 and Sortilin through protein docking assays, we first obtained the crystal structure of the two proteins from the MICOS platform (Fig. 4A, B). Blinding docking analysis results implied that indeed Presenilin1 and Sortilin interacted. The RMSD analyses indicated a stable state after 60 ns and obtained the lowest energy docking model, and the lowest energy of molecular docking was − 83.55 kcal/mol (Fig. 4C). We displayed the different angle views of the Presenilin1-Sortilin docking model in Fig. 4D–G, and the interaction between Presenilin1-Sortilin was focused on the transmembrane domain. The interface was a relatively close hydrophobic binding system formed by hydrophobic amino acids (Fig. 4H). The key interacting residues of amino acids in the dimer interface from Sortilin were V757, L761, P758, A762, V769, A773, L776, L768 and I777, and those from Presenilin1 were V125, W244, L248, F447, Y451, A251, I439, V255, V259, C263, P264, L262, P428 and P436 (Fig. 4I–K).

### Sortilin is a substrate for Presenilin1 and mediates the suppression of Presenilin1 on β-catenin in glioblastoma cells

**Fig. 5:**
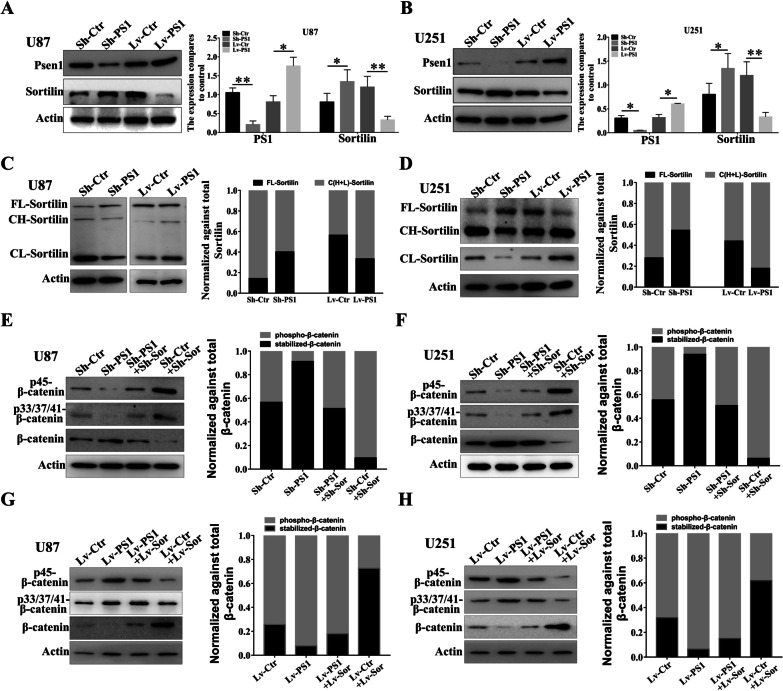
Sortilin is substrate for Presenilin1 and mediates the suppression of Presenilin1 on β-catenin in glioblastoma cell. **A**, **B** Western blot analysis the expression change of Sortilin inU87 and U251 cells transfected with Lv-PS1, Sh-PS1 or control. **C**, **D** Western-blot assays to investigate the expression levels of full length (FL-) Sortilin, cleavage heavy (CH-) Sortilin and cleavage light (CL-) Sortilin after down- or up-expression ofPresenilin1 in U87 and U251 cell. **E**, **F** Western-blot assays to show the expression ofp45-β-catenin, p33/37/41-β-catenin, stabilized β-catenin when down-regulation of Presenilin1 or combined with down-regulation of Sortilin in U87 and U251 cells. **G**, **H** Western-blot assays to show the expression ofp45-β-catenin, p33/37/41-β-catenin, stabilized β-catenin when up-regulation of Presenilin1 or combined with up-regulation of Sortilin in U87 and U251 cell. **p < 0.01, *p < 0.05

According to the close relationship of Presenilin1 and Sortilin, we investigated the deep interaction mechanism. As shown in Fig. 5A, downregulation of Presenilin1 sharply increased the protein expression of Sortilin; inversely, overexpression of Presenilin1 apparently decreased the protein expression of Sortilin in U87 cells. We repeated the above assay in U251 cells and obtained similar results (Fig. 5B). However, we did not find an obvious change in Presenilin1 protein whether Sortilin was overexpressed or downregulated in glioblastoma cells (data not shown). Sequentially, we extracted whole cell lysates for western blotting. Two Sortilin proteins with lighter molecular weights (approximately 16 kDa and 95 kDa) were detected in U87 cells in addition to full-length Sortilin (110 kDa). According to a previous report [17], we recognized two lighter blots as cleavage Sortilin in U87 cells. Furthermore, we found that downregulation of Presenilin1 increased the expression of full-length Sortilin; however, the cleavage of Sortilin was decreased when Presenilin1 was repressed in U87 cells. In contrast, overexpression of Presenilin1 decreased full-length Sortilin and increased the cleavage of two Sortilin proteins in U87 cells (Fig. 5C). Similarly, we repeated the above assays in U251 cells and observed the same trends after loss- or gain-of-function of Presenilin1 (Fig. 5D), which implies that Presenilin1 regulates Sortilin cleavage in glioblastoma cells. Our previous research demonstrated that both Sortilin and Presenilin1 target β-catenin in glioblastoma. Thus, we also detected whether Sortilin influenced the function of Presenilin1 on the phosphorylation of β-catenin. As expected, we found that the phosphorylation of β-catenin at sites 45 and 33/37/41 was sharply decreased, accompanied by an increase in stabilized β-catenin after Presenilin1 inhibition. Furthermore, phosphorylated β-catenin was recovered when Sortilin was downregulated in U87 cells (Fig. 5E). Similar results were observed in U251 cells (Fig. 5F). In contrast, the phosphorylation levels of β-catenin were significantly elevated and the stabilized β-catenin was clearly reduced after Presenilin1 stimulation, and overexpression of Sortilin alleviated the phosphorylated/stabilized ratio of β-catenin in U87 and U251 cells stimulated with Presenilin1 (Fig. 5G, H). These results indicate that Presenilin1 cleaves Sortilin and that Sortilin mediates β-catenin phosphorylation-degradation by Presenilin1 in glioblastoma cells.

### Presenilin1 represses MT and invasion though the Sortilin/β-catenin axis in glioblastoma cells

**Fig. 6:**
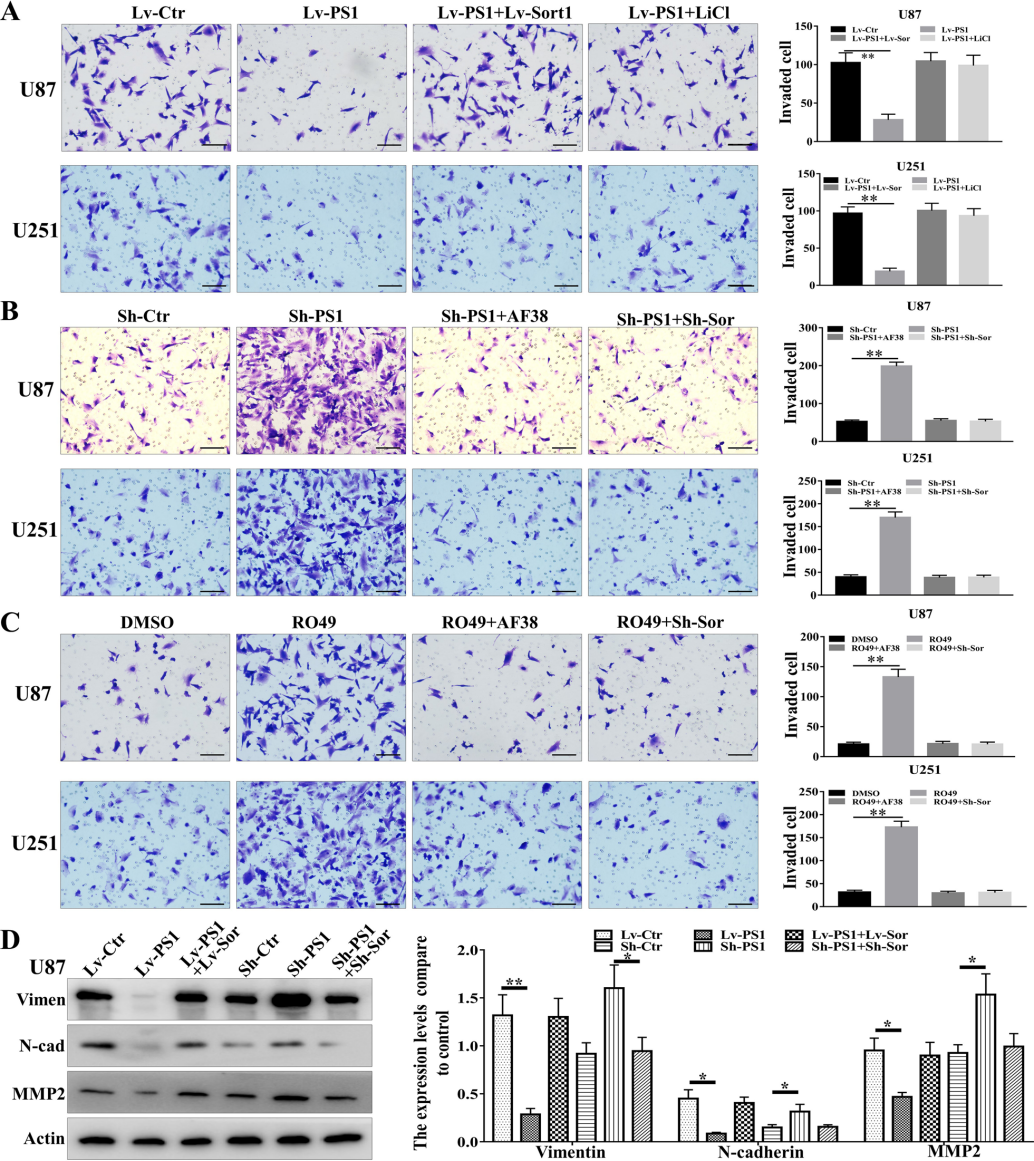
Presenilin1represses MT and invasion though Sortilin/β-catenin axis in glioblastoma cell. **A** U87 and U251 cells were treated with Lv-PS1 alone, combined with Lv-Sort1 or with LiCl (Sortilin/β-catenin axis activator). Invaded cells were stained and counted using microscopy. **B** Transwell assays to investigate the invasion ability when down-regulation of Presenilin1, combined with Sortilin inhibitor AF38469 or with down-regulation of Sortilin in U87 and U251 cell. **C** Transwell assays to detect the invasion ability of U87 and U251 cells when treated with RO4909497 alone, combined with AF38469 or with down-regulation of Sortilin in U87 and U251 cell. **D** Western blot analysis of the expression levels of MT markers (N-cadherin, vimentin, MMP-2) in indicated groups of U87cells. Scale bar = 100 μm.**p < 0.01, *p < 0.05

The above results imply that Presenilin1 enhances the cleavage of Sortilin and then inhibits downstream β-catenin in glioblastoma cells. We redetected the expression of Sortilin in glioblastoma tissues and found that Sortilin was highly expressed in tumor cells at the margin area, implying that Sortilin plays a vital role in promoting glioblastoma cell invasion (Additional file 3: Figure S2J). Next, we detected whether Sortilin/β-catenin mediates the anti-invasion function of preseninlin1. First, we overexpressed the Presenilin1 gene and observed a clear decrease in invaded cells; however, combining overexpression of Sortilin1 rescued the depression of invasiveness caused by Presenilin1 stimulation. Moreover, treatment with an activator of the Sortilin/β-catenin axis (LiCl) also recovered the invasion ability of U87 and U251 cells treated with Presenilin1 (Fig. 6A). Conversely, Presenilin1 silencing sharply increased the number of invaded cells, but the invasiveness was abrogated when combined with Sortilin inhibitor AF38469 treatment. Additionally, SORT1 gene knockdown also remitted the promotion of invasion when presenlin1 was repressed in U87 and U251 cells (Fig. 6B). Moreover, the Presenilin1 inhibitor RO49 also increased the invasion ability of glioblastoma cells, and both downregulation of Sortilin and AF38469 addition alleviated the pro-invasion caused by Presenilin1 suppression (Fig. 6C). Furthermore, to investigate whether Sortilin also transduces the anti-MT function of Presenilin1 in glioblastoma cells, we employed western blotting assays to detect Vimentin, N-cadherin, and MMP2 expression when cells were lost or gained function of Presenilin1 and/or Sortilin. Sortilin activation relieved the inhibition of the MT process after Presenilin1 stimulation, and Sortilin repression also recovered the stimulation of the MT process after Presenilin1 suppression in U87 and U251 cells (Fig. 6D, Additional file 4: Figure S3A). The above results illustrate that Presenilin1 exerts anti-MT and anti-invasion effects through Sortilin in glioblastoma cells.

### Presenilin1 suppresses MT and invasion of glioblastoma in vivo

**Fig. 7:**
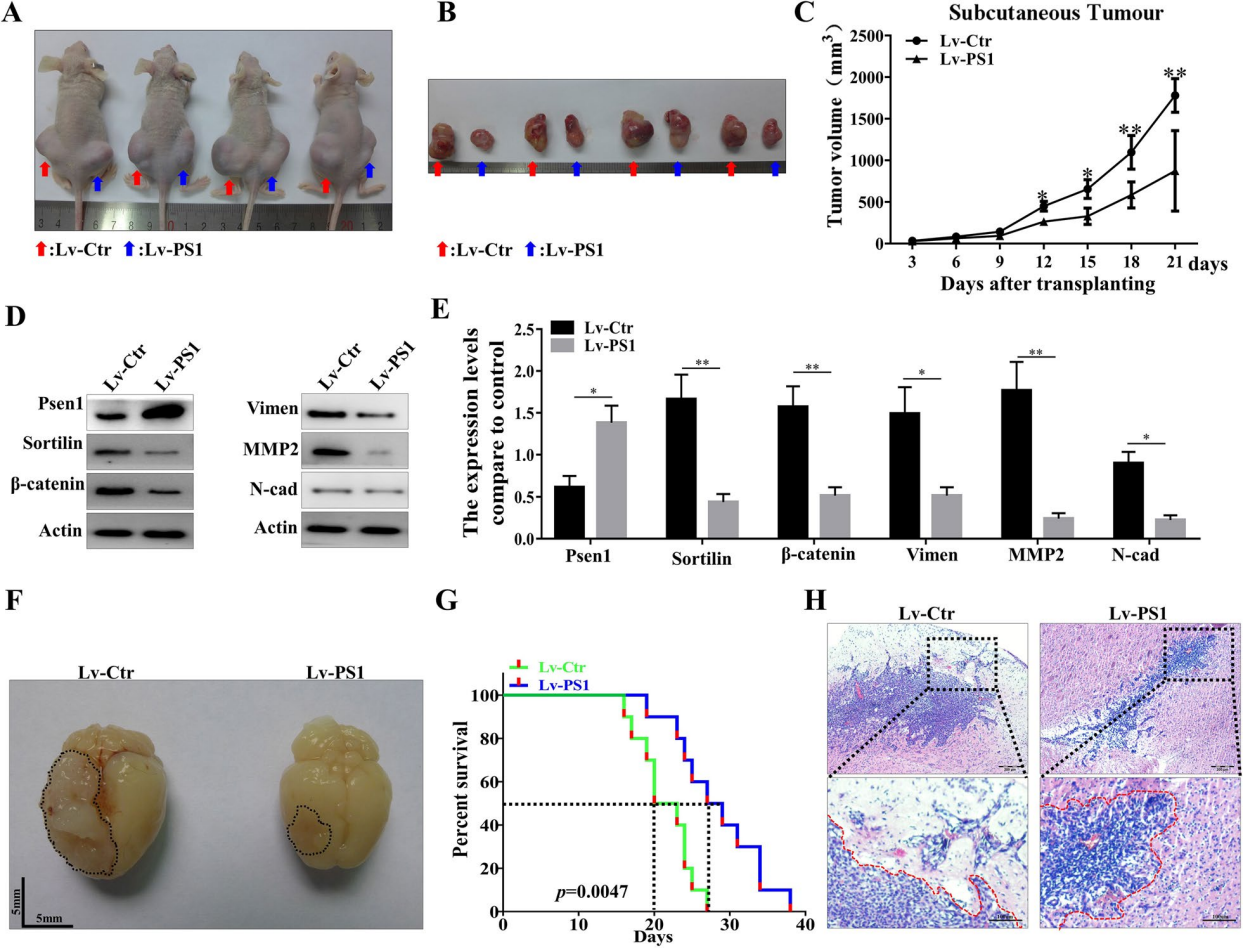
Presenilin1 suppress MT and invasion of glioblastoma in vivo. **A–C** The gross observation of subcutaneous glioblastoma at day 21 post injection when over-expression of Presenilin1 or control, the volume of tumor was measured and calculated every three days after transplanting. **D** Western blot analysis of the activation of Sortilin/β-catenin axis and the expression levels of MT markers (N-cadherin, vimentin and MMP-2) in subcutaneous glioblastoma tissues. **E** Representative gross observations (scale bar = 5 mm) of orthotopic glioblastoma xenografts in the indicated groups. **F** The survival time of glioblastoma-bearing mice was analyzed by Kaplan–Meier method and compared by log-rank test. **G** H&E staining to show the invasion-growth states of orthotopic xenografts, dotted black line to show the border of glioblastoma. Scale bar = 50 μm. *p < 0.05, **p < 0.01

To further verify the effects of Presenilin1 on glioblastoma invasion and mesenchymal transition in vivo, we subcutaneously implanted 1 × 10^6^ U87-Lv-Ctr and U87-Lv-PS1 cells into the right and left groins of nude mice. There was a significant difference between the two groups of tumor values. Overexpression of Presenilin1 clearly retarded the invasive growth of glioblastoma cells (Fig. 7A, B). After 21 days, the tumor volume of subcutaneous tumors in the Lv-PS1 group was sharply smaller than that in the Lv-Ctr group (Fig. 7C), indicating that Presenilin1 stimulation attenuates subcutaneous GBM invasion-growth. By western blotting, we found that overexpression of Presenilin1 suppresses the Sortilin/β-catenin axis and MT process in glioblastoma tissues (Fig. 7D–E). Next, we established orthotopic GBM xenograft models by implanting U87 cells into the right striatum of naked mice. The results indicated that Presenilin1 overexpression significantly decelerated the invasion of orthotopic xenografts (Fig. 7F). Kaplan–Meier survival analysis showed that the median survival time of mice in the Lv-PS1 group (28.0 days) was significantly longer than that of mice in the DMSO group (21.5 days) (Fig. 7G). In addition, the results of HE staining of transplanted GBM tissues showed that the tumor values of the Lv-PS1 group were smaller than those of the control group. Foremost, the tumor margin of the Lv-PS1 group was clearer and smoother than that of the DMSO group, suggesting that Presenilin1 suppresses the invasive capacity of intracranial glioblastoma cells (Fig. 7H). These data confirm that Presenilin1 prevents GBM invasion possibly via Sortilin/β-catenin-induced mesenchymal transition in vivo, while stimulation of Presenilin1 impairs GBM invasion and mesenchymal transition and prolongs the survival time of GBM-bearing mice.

**Fig. 8:**
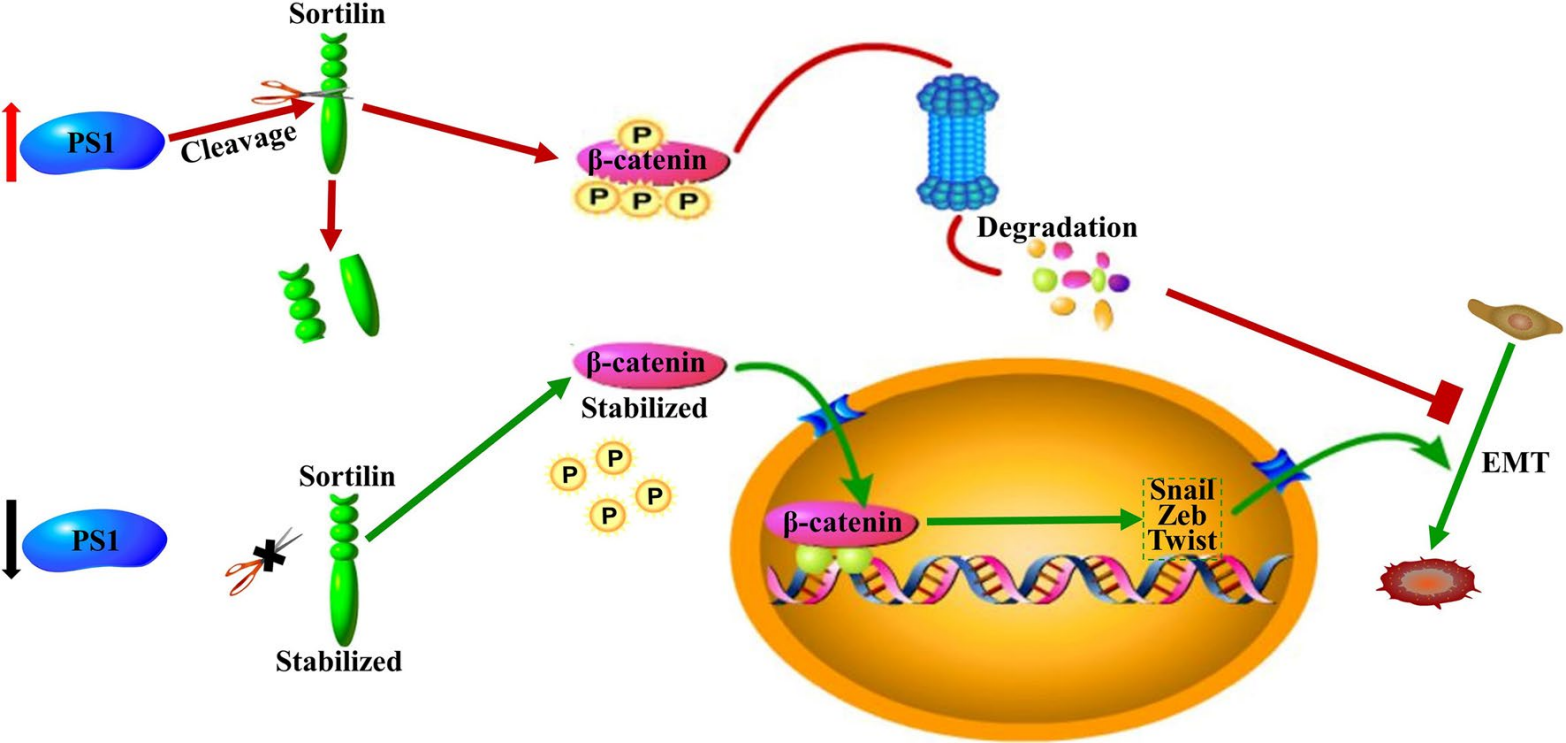
Schematic model. Presenlin1 represses mesenchymal transition and invasion target β-catenin phosphorylation trough cleaving Sortilin in human glioblastoma cell

Collectively, our data demonstrated that Presenilin1 and Sortilin are critical partners with opposite effects in glioblastoma cells. Specifically, we observed that Presenilin1 deficiency in high-grade glioma maintained persistent levels of cleaved Sortilin and promoted phosphorylation-degradation of β-catenin, while Presenilin1 repression released Sortilin and stabilized β-catenin, enhancing mesenchymal transition and increasing glioblastoma cell invasiveness (Fig. 8).

## Discussion

The identification of molecular involved in glioblastoma mesenchymal transition and aggressiveness is of high interest for cancer research [19]. And our previous research suggests that Alzheimer disease protein-Presenilin1 acts as anti-proliferation role in glioblastoma cell [7]. Besides proliferation effect, the diffuse invading behave of glioblastoma cell also makes tumor to be lethal most [2]. Thus, we wondered whether Presenilin1 participated in glioma invasiveness and we aimed to identify the relative mechanism involved. Here, we obtained that Presenilin1 exerts anti-invasion function through Sortilin/β-catenin axis, and identified Sortilin as mediator to transducing Presenilin1 regulation on β-catenin phosphorylation-degradation in glioblastoma cell. Our research deeply enhances the impression of anti-glioma function of Presenilin1 and furtherly explains why glioma is barely found in AD patients.

Alzheimer’s disease (AD) is one of the most common neurodegenerative diseases, which is clinically manifested as memory loss, cognitive decline, personality change and various neurological symptoms [20].Recently, although little is known about the cause of Alzheimer’s disease and no curative treatments are available, a growing number of premising molecular were detected in the past decade [21]. Among them, Presenilin1 participates in APP cleavage by γ-secretase of AD pathogenesis. At present, Presenilin1 provide the catalytic protease activity that is independent and necessary for γ-secretase [22].The mutations and deletion of preseilin1 are reported to cause AD (especially in early-onset AD) through aberrant APP processing determining either increased Aβ levels or increased production of Aβ42 (and Aβ43) peptide rather than Aβ40, leading to Aβ aggregation [23]. Besides, Presenilin1 also cleaves another type 1 transmembrane protein such as Alcadein α, Deleted in colorectal cancer (DCC), LDL receptor family (LRP1 and LRP8), p75-neurotrophin receptor (p75NTR), NOTCH receptor [6]. Recently, several observations strongly suggest that the Vps10p receptors family (contains Sortilin, SorCS1, SorCS2, SorCS3, and SorLA) also is identified to be the substrate of Presenilin1 [17]. Sortilin was reported to regulating the processing and trafficking of amyloid precursor protein with another type 1receptor SorLA, detailly, Sortilin mediates the trafficking of APP into lysosomes and lipid rafts in neurites of hippocampal neurons. The presence of Sortilin promotes α-secretase cleavage of APP, unlike SorLA, which inhibits the generation of all soluble products [24, 25]. Here, we found Presenilin1 interacted with Sortilin in glioblastoma, which may expand the mechanism of AD, and Presenilin1 interaction with Sortilin may play crucial role in AD and neuronal death. Additionally, P75^NTR^ is another substrate for Presenilin1 dependent γ-secretase [26], itis well investigated that Sortilin interacts with P75^NTR^ transduces the function of proNGF or ProBDNF to exerts neuronal death signaling [27]. Therefore, additional studies are needed to clarify the role of Presenilin1 on pro-Neurotrophin/Sortilin-P75^NTR^ axis in physiologic and possible pathologic process.

JP Roperch, etl firstly reports Presenilin1 is downregulated in a series of model systems for p53-dependent and p53-independent apoptosis and tumor suppression, and down-expression of Presenilin1 results in reduced growth with increased apoptosis in U937 cells, suggesting Presenilin1 acts as promotor in tumorigenesis [28]. Next opposite study demonstrates presenilin deficiency results in increased beta-catenin stability in vitro and in vivo, this investigator recognized presenilin as anti-tumor molecular in medulloblastomas [5]. Following studies demonstrated Presenilin1 participates in cancer dominating focus on γ-secretase cleavage. Notch receptor is the second Presenilin1 substrate identified after APP [29], it is cleaved by Presenilin1 and releases into the cytoplasm its intracellular domain, which is a well-known transcription factor for several genes, mostly involved in tumor cell survival and differentiation [30]. Similarly, CD44 is up-expressed in subpopulations of cancer cells and recognized as a molecular marker for cancer stem cells. Presenilin1 mediates the intramembranous cleavage of CD44 to influencing tumor cell growth and metastasis [8, 31]. Recently, Presenilin1exerts inverse function was reported in leukemia, malignant mesothelioma, lung cancer, hepatocellular carcinoma, bladder cancer [32–36]. Here, our investigation only finds the anti-proliferation and anti-invasion role of Presenilin1 in glioma.

Kang et al. and our previous study demonstrates that Presenilin1 represses β-catenin axis by phosphorylated and decreased the stability of β-catenin in tumorigenesis [5]. However, the detail of regulation mechanism of Presenilin1 on β-catenin was not clearly expounded. Recently, Cadherin is reported to connect the preseninlin1 to β-catenin. On one hand, Presenilin1forms complexes with both E- and N-cadherin and concentrates at synaptic adhesions, and also with cadherin/catenin adhesion system and regulates cell–cell adhesion in MDCK cells [16]. On the other hand, The Presenilin1/gamma-secretase system stimulates disassembly of the E-cadherin-catenin complex to release the cytoplasmic E-cadherin to the cytosol and increases the levels of solubleβ- and α-catenin [15, 37].Recently, it is generally acknowledged that the source of glioma cell was not from epithelial cells. And the expression of E-cadherin is deficiency in major glioma cells [2]. In our study, we didn’t detect the expression of E-cadherin in U87 and U251 cells. T-cadherin as a substitution for E-cadherin in glioma was not significantly change in lose- or gain- function of Presenilin1 (data not shown). Here, we identified Sortilin transduced Presenilin1 anti-invasion effect by promoting β-catenin phosphorylation and degradation, leading to decreasing MT-transcription factors and repressing MT process in glioblastoma cells. Our previous results manifest that Sortilin plays a pivotal role in stabling β-catenin and preventing it to be degraded in proteases [11]. In this study, we further confirmed that full length Sortilin is the prerequisite for β-catenin stabilization and downstream signaling activation. However, the cleavage of Sortilin strongly eliminates its protection for β-catenin degradation. Which implying Presenilin1/Sortilin/β-catenin axis is a vital pathway in anti-invasion of glioma.

In this study, we focused on the Presenilin1/Sortilin/β-catenin axis, because it is well investigated thatβ-catenin/ EMT transcription factors as the most important signaling pathway in the mesenchymal transition of GBM [38]. However, one limitation of our study is that the accurate cleavage site of Sortilin by Presenilin1was not investigated in this study. Previous research implies that Sortilin is cystein-rich protein belong to type I single transmembrane receptor. Sortilin is synthesized as a proform which is converted to the mature receptor by furin-mediated cleavage of a 44 residue N-terminal propeptide in late Golgi compartments [39]. And spadin, a secreted peptide derived from the propeptide generated by the maturation of Sortilin exerts putative antidepressant function [40]. Furthermore, Sortilin could shed the extramembrane luminal domain and release endogenous receptor in HT29 cell, and the shedding and soluble receptor form of the Sortilin is able to bind Neurotensin [41]. Following study illustrates Presenilin1 dependent γ-secretase cleaves Sortilin after the shedding of ectodomain and releases 16 KDa COOH-terminal fragment [17],which is consist with our research, but the cleavage site still needs to be investigated in further. In addition, although we found intact Sortilin was crucial for activation of β-catenin, the detail mechanism of Sortilin in mediating β-catenin still unclear. Considering to Sortilin could heterodimerize with GPCR receptor, such as NTSR1 and EGFR, and influence downstream activation [42, 43], we suspect Sortilin may interact with or modify Frizzled receptor of WNT pathway. On the other hands, Sortilin also mediates a lot molecular trafficking and secretion in cancer cell [8], whether Sortilin regulates β-catenin by influencing WNTs production and transport also needs to be explored.

## Conclusions

These results suggest that Stimulation of Presenilin1 inhibited glioblastoma invasiveness in vitro and in vivo. Furthermore, type 1 receptor Sortilin functions as the downstream targets of Presenilin1 to regulate β-catenin-dependent mesenchymal transition and invasion. Our findings revealed the putative mechanism for Presenilin1in tumor cell invasion. We also revealed that the presenilin/Sortilin/β-catenin signaling axis may be a novel target for the treatment of glioblastoma.

## Supplementary Information

**Additional file 1**. **Figure S1**. **A:** Kaplan–Meier analysis for all grade glioma patients. patients in the high Presenilin1 group (n = 11) and in the low Presenilin1 group (n = 11) (*p* = 0.412, log-rank test). **B:** IHC assays to detect the expression of Presenilin1 in human glioblastoma tissues, Scale bar = 50 μm and 20 μm (inset image), respectively. **C:** the knockdown and over-expression effect of lentivirus contains sh-PS1 and lv-PS1. **D:** Representative images of wound healing assays using U251 cells after down-expression of Presenilin1. **E:** Western blot were performed to analysis the expression change of mesenchymal markers (Vimentin, N-cadherin and MMP2) inU87 and U251 cells when treated with Presenilin1 inhibitor RO4909497(RO49). *p < 0.05, **p < 0.01.

**Additional file 2**. **Figure S2**. **A-B:** The correlations of Presenilin1 with mesenchymal transition transcription factors (Slug1 and Slug2) from French-284-glioma dataset. **C:** The interaction of Sortilin and related proteins with performed on Pathway commons platform. **D-F:** The relations of Presenilin1 with Sortilin in each WHO grades primary glioma (Grade II, III, IV)from mRNAseq_693 dataset on CGGA. **G-I:** The relations of Presenilin1 with Sortilin in each WHO grades recurrent glioma (Grade II, III, IV)from mRNAseq_693 dataset on CGGA. **J:** The expression pattern of Sortilin in the peri-tumor of human glioblastoma tissue. Nestin is used to label tumor cell. Scale bar = 5 μm. The R and *p* values were downloaded and shown. *p < 0.05, **p < 0.01.

**Additional file 3**. **Figure S3**. **A:** Western blot analysis of the expression levels of MT markers (N-cadherin, vimentin, MMP-2) in indicated groups of U87cells. *p < 0.05, **p < 0.01.

## Data Availability

The data used or analyzed during this study are included in this article and available from the open access website or platform.
