# Supplementary figures and images for: Presenilin1 inhibits glioblastoma cell invasiveness via promoting Sortilin cleavage
Source: Cell Commun Signal. 2021 Nov 15;19:112. doi: 10.1186/s12964-021-00780-5 (PMC8594175; doi:10.1186/s12964-021-00780-5)

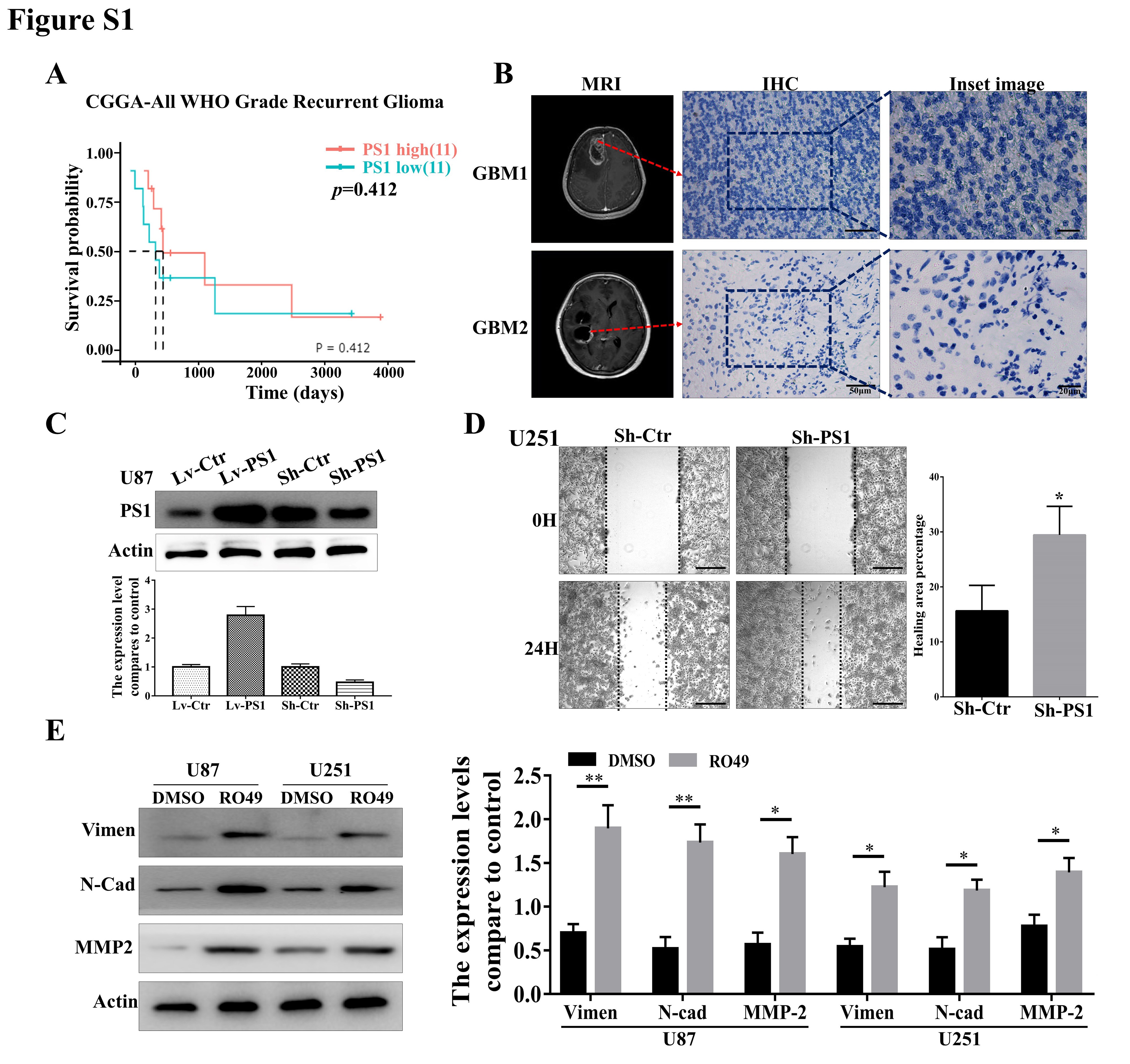

Supplement: Supplementary file 2 — Additional file 1. Figure S1. A: Kaplan–Meier analysis for all grade glioma patients. patients in the high Presenilin1 group (n = 11) and in the low Presenilin1 group (n = 11) (p = 0.412, log-rank test). B: IHC assays to detect the expression of Presenilin1 in human glioblastoma tissues, Scale bar = 50 μm and 20 μm (inset image), respectively. C: the knockdown and over-expression effect of lentivirus contains sh-PS1 and lv-PS1. D: Representative images of wound healing assays using U251 cells after down-expression of Presenilin1. E: Western blot were performed to analysis the expression change of mesenchymal markers (Vimentin, N-cadherin and MMP2) inU87 and U251 cells when treated with Presenilin1 inhibitor RO4909497(RO49). *p < 0.05, **p < 0.01. [file 12964_2021_780_MOESM2_ESM.jpg]

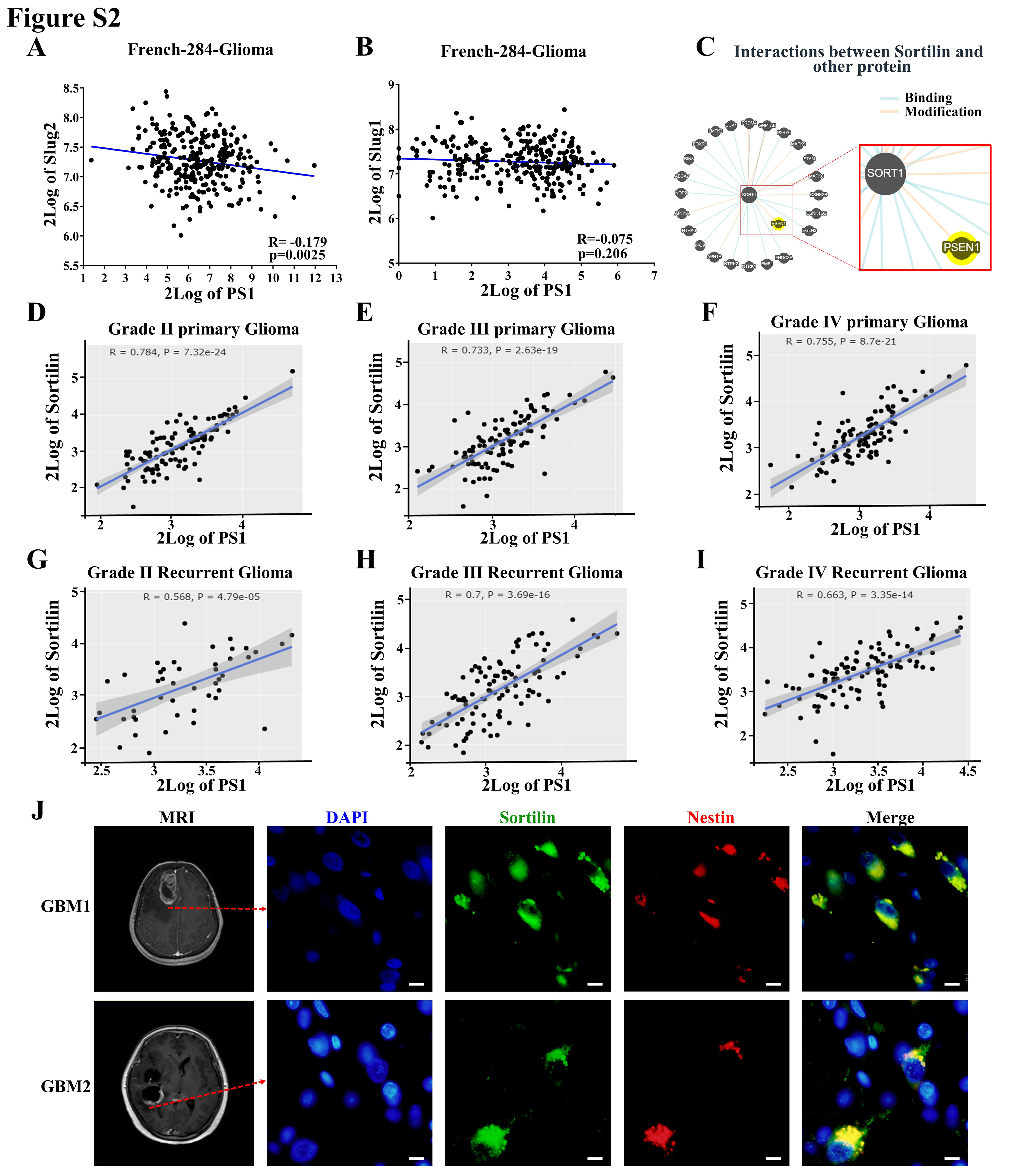

Supplement: Supplementary file 3 — Additional file 2. Figure S2. A-B: The correlations of Presenilin1 with mesenchymal transition transcription factors (Slug1 and Slug2) from French-284-glioma dataset. C: The interaction of Sortilin and related proteins with performed on Pathway commons platform. D-F: The relations of Presenilin1 with Sortilin in each WHO grades primary glioma (Grade II, III, IV)from mRNAseq_693 dataset on CGGA. G-I: The relations of Presenilin1 with Sortilin in each WHO grades recurrent glioma (Grade II, III, IV)from mRNAseq_693 dataset on CGGA. J: The expression pattern of Sortilin in the peri-tumor of human glioblastoma tissue. Nestin is used to label tumor cell. Scale bar = 5 μm. The R and p values were downloaded and shown. *p < 0.05, **p < 0.01. [file 12964_2021_780_MOESM3_ESM.jpg]

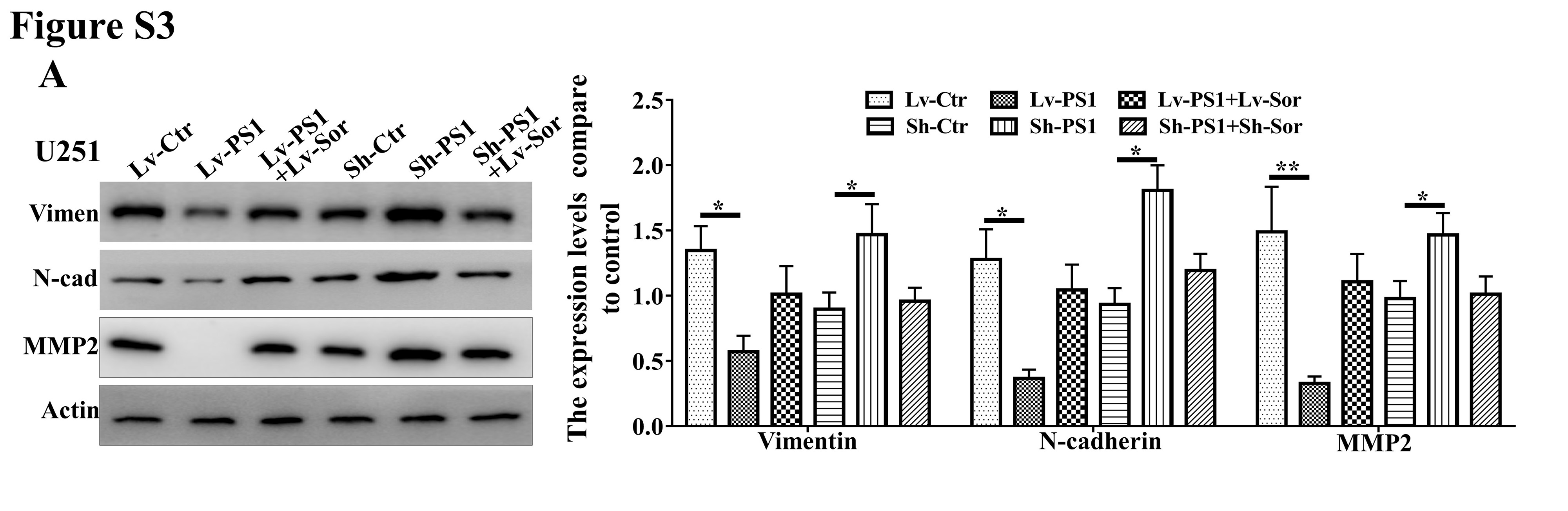

Supplement: Supplementary file 4 — Additional file 3. Figure S3. A: Western blot analysis of the expression levels of MT markers (N-cadherin, vimentin, MMP-2) in indicated groups of U87cells. *p < 0.05, **p < 0.01. [file 12964_2021_780_MOESM4_ESM.jpg]
